# A Tale of 2 Countries: Implementation of the Cold Chain Equipment Optimization Platform in Guinea and Kenya

**DOI:** 10.9745/GHSP-D-22-00066

**Published:** 2022-10-31

**Authors:** Emily Stammer, Lea Teklemariam, Aliou Barry, Roger Millimono, Amos Chweya, Nicole Danfakha, Caddi Golia, Elena Herrera, Leslie Patykewich, Wendy Prosser, Soumya Alva

**Affiliations:** a JSI Research & Training Institute, Inc., Arlington, VA, USA.; b Stat View International, Conakry, Guinea.; c Independent Consultant, Conakry, Guinea.; d JSI Research & Training Institute, Inc., Nairobi, Kenya.

## Abstract

In 2016, the Gavi Cold Chain Equipment Optimization Platform (CCEOP) was approved and launched in recognition of the fact that functional cold chain equipment (CCE) is essential to strengthening vaccine supply chains and ultimately achieving Gavi’s immunization equity and coverage goals. Through CCEOP, Gavi committed to investing US$250 million between 2016 and 2021 to commission CCE in more than 63,000 facilities to upgrade and expand their CCE footprint while stimulating the market to provide affordable, technologically advanced, and accessible equipment. We present case studies from Guinea and Kenya, both of which received CCEOP support, that highlight 2 ways for countries to prioritize investments and implement activities through a large funding and support mechanism. The studies explore the different ways that each country implemented CCEOP and consider how aspects of leadership and technical capacity influence country priorities and results. They also uncover key lessons on sustainability of a large immunization supply chain effort. The experiences of Guinea and Kenya can help other countries embarking on similarly large health system interventions, especially related to supply chain strengthening and immunization programs. In particular, these experiences offer important lessons in leadership, processes and systems, country ownership, technical capacity, and sustainability.

## Abstract (precis)

Case studies of Gavi Cold Chain Equipment Optimization Platform investments in Guinea and Kenya illustrate how countries prioritize and implement large funding and support mechanisms and offer lessons applicable to other countries embarking on similar interventions.

## Key Messages

- Implementation of a health system intervention involving immunization requires strong country leadership and ownership in the form of experienced logistics professionals and knowledgeable, dedicated staff in national vaccine and immunization programs.
- To maximize efficiencies, the Gavi Cold Chain Equipment Optimization Platform and other initiatives of similar size require coordination and alignment with ministry systems and processes as well as those of other funder/partner initiatives.
- Outsourcing a specific function such as installation of equipment to a third-party provider can benefit countries, but governments need to analyze the cost benefit of outsourcing to providers and the skills and systems required to manage and coordinate with them.
- Cold chain equipment strengthens the vaccine supply chain and improves immunization equity and coverage, but its continued sustainability as an investment in the health system depends on ongoing resources to maintain it. Without assuring operational and financial sustainability, unreliable cold chain systems dependent on external funding and the immunization programs that rely on them will remain unimproved.

Résumé en français à la fin de l'article.

## INTRODUCTION

In 2019, more than 5.2 million children around the world died before their fifth birthday, mostly from preventable causes.^1^ Immunization has been recognized as 1 of the most successful public health interventions,^2^ with estimates that it could prevent 2 to 3 million deaths per year.^3^ Despite this, global vaccination rates remained stagnant at 85% for the past several years and fell to 83% in 2020.^3^ Availability of effective and appropriate cold chain equipment (CCE) supports efforts to immunize all children, including those in remote, hard-to-reach locations where immunization coverage can easily falter. The maintenance and management of CCE to ensure vaccine potency are also essential for improving immunization coverage.^4^

### Cold Chain Equipment Optimization Platform

Gavi, the Vaccine Alliance’s Cold Chain Equipment Optimization Platform (CCEOP) was established in 2015 and approved and launched in 2016, in recognition of the fact that functional CCE is a critical precondition to strengthening vaccine supply chains and ultimately achieving Gavi’s immunization equity and coverage goals.^5^ Through CCEOP, Gavi committed to investing more than US$250 million between 2016 and 2021 in more than 63,000 facilities globally to upgrade and expand the cold chain capacity while stimulating the market to provide affordable, technologically advanced, and accessible equipment.^6^^,^^7^ This approach is guided by Gavi’s Immunization Supply Chain Strategy, which provides an end-to-end perspective of the supply chain and emphasizes the 5 supply chain fundamentals of leadership, continuous improvement and planning, data for management, CCE, and system design.^8^

CCEOP consists of 2 main approaches. The global-level, market-shaping approach seeks to improve the availability and installation of high-performing CCE, underscoring the need to ensure a healthy global market for CCE in which countries are procuring durable and high-performing products. The second approach focuses on country-level implementation to upgrade and expand CCE, creating a more efficient and effective supply chain.

CCEOP seeks to improve efficiencies during new equipment deployment and to strengthen ministry of health (MOH) and Expanded Program on Immunization (EPI) management processes to support country ownership. Gavi and UNICEF have created processes for coordination and collaboration and developed standard systems and requirements to support each CCEOP partner country and strengthen the management capacity of EPI leaders. For example, Gavi requires all recipient countries to establish a program management team (PMT) to oversee CCEOP planning and implementation; conduct a cold chain inventory to inform CCE selection; and develop an operational deployment plan (ODP) to guide CCE placement.

CCEOP seeks to improve efficiencies during new CCE deployment and to strengthen MOH and EPI management processes to support country ownership.

Once applications to Gavi are approved, UNICEF procures CCE at globally negotiated rates from an approved list of manufacturers for respective countries based on their requirements. In-country private service bundle providers (SBPs) selected by the manufacturers install and maintain the equipment during the warranty period. For countries in the first phase of investments, like Guinea and Kenya, contracts with local SBPs were required. In later phases, countries could opt out or delink from the SBPs. UNICEF pays the SBP contracts, while the respective country PMT and UNICEF country office are responsible for approving their installation reports as a prerequisite for receiving payment. The CCEOP framework included this approach to capitalize on the efficiencies of the private sector while developing the supply chain system in countries.^9^ As part of its contracts, Gavi also required that the SBPs train cold chain technicians and health workers in country health facilities on maintenance to ensure continuity after the warranties for specific CCE models end.

As part of CCEOP’s first phase, Guinea and Kenya received support in 2017 to obtain CCE for expansion and extension of their immunization supply chains. In this context, expansion refers to replacing or upgrading old, obsolete, or inadequate CCE in facilities currently offering immunization services, and extension refers to equipping facilities not currently offering immunization services or only offering them as part of outreach with new CCE. The Table provides an overview of the major activities in each country. The experiences of Guinea and Kenya highlight how countries can prioritize and implement large funding and support mechanisms, which can help other countries embarking on similar interventions, especially related to supply chain strengthening and immunization programs.

**TABLE.** Cold Chain Equipment Optimization Platform Activities in Guinea and Kenya

	**Guinea**	**Kenya**
Date of CCEOP approval	October 2017	March 2017
Amount approved, US$	10.9 million^a^	8.2 million^a^
Financing by country, %	20	50
Equipment models	3 (2 solar powered and 1 passive vaccine storage device [no power source])	8 (3 solar powered and 5 electric powered)
Deployment 1 priority	Equipping health facilities to expand immunization services by increasing the number of fixed vaccination locales	Replacing^b^ equipment in facilities with storage gaps in all counties and equipping new facilities in 17 HSS-priority counties
Deployment 2 priority	Replacing equipment in health centers with non-functioning CCE	For those not covered in Deployment 1, continue re-placing equipment in facilities with storage gaps and providing CCE in facilities without it
Time frame		
Deployment 1	December 2018–June 2019^a^	July–December 2018^a^
Deployment 2	November 2021–May 2022 (delayed)	June–December 2021 (delayed)

Abbreviations: CCE, cold chain equipment; CCEOP, Cold Chain Equipment Optimization Platform; HSS, health systems strengthening.

a Unpublished data.

b In cases where equipment existed and was replaced to address storage gaps, replacement equipment had greater storage capacity than existing equipment.

The case studies presented here explore: (1) ways in which each country implemented CCEOP; (2) how aspects of country leadership and human resource capacity influenced priorities and results; and (3) key lessons in sustainability of national programs investing in the immunization supply chain. These 3 topics were selected based on their critical importance to CCEOP success and because the experiences from Guinea and Kenya offered the most insights around these themes. We do not delve into issues related to financing or national policies, which also have roles.

## METHODS

The case studies draw upon a multicountry prospective mixed-methods evaluation of CCEOP conducted between 2018 and 2021 that explored CCEOP design components and implementation processes. The evaluation was led by JSI Research & Training Institute, Inc. (JSI) in conjunction with local partners (Stat View International in Guinea; JaRco Consulting and InSupply Health in Kenya). The evaluation consisted of a health facility assessment (HFA) conducted between January 2018 and March 2021 at 4 time points in Kenya and 3 time points in Guinea, as well as key informant interviews with national and subnational-level stakeholders. The HFA provides a snapshot of the status of CCE in a small set of facilities, with information on functionality, stock management procedures, and immunization service provision.

The sample size for the HFA was based on information in the ODP and covered facilities in selected regions/counties, some of which received CCE as part of the deployment (program facilities) and some that did not (control facilities). In Kenya, the HFA included a mix of program and control facilities in each of the 3 counties (Homa Bay, Kitui, and Marsabit), while in Guinea program facilities were in Boké and Faranah. Kankan, which received no CCE, served as the control for Guinea.

Excepting a few adjustments due to deployment changes, the sampled health facilities identified at baseline remained the same through endline. In Guinea, 110 facilities (health centers and posts) and 12 district depots in the regions were included in the HFA sample. At the time of sampling, the sample of facilities made up 26% of health posts and health centers in the 3 regions. All district depots in the 3 regions were represented in the sample. Additionally, the team conducted 72 key informant interviews at endline with partners, government officials, and SBPs at the national level, MOH officials including cold chain technicians at the regional and district levels, and staff at health centers and health posts in the 3 regions. This analysis focuses on health posts in Guinea, in correlation with the first CCE deployment.

In Kenya, the endline HFA sample was 136 health facilities and 13 subcounty stores, for a total of 149 facilities from the 3 counties. The sample of health facilities made up approximately 19% of health facilities in the 3 counties and 65% of subcounty stores at the time of sampling. The research team also conducted 63 interviews with government officials, partner organizations, and SBPs at the national level, and MOH officials including cold chain technicians at county, subcounty, and facility levels.

The evaluation also included a secondary analysis of program documentation including the CCEOP application, operational deployment plans, monitoring documents, and PMT notes, where available. JSI sent data collection tools to MOH representatives from both countries at each time point and incorporated feedback and obtained written approval before each data collection. The findings we present are drawn from the baseline and endline evaluations. Given the timing of the equipment deployments (additional deployments were planned but delayed in both countries), the evaluation focused on priorities and activities of the first deployment.

### Ethical Approval

The evaluation was classified as exempt from review by the JSI Institutional Review Board since it involved survey activities without identifiers or sensitive questions that could result in harm.

## RESULTS

Each case study highlights findings relevant to planning, implementing, and managing large health system interventions and provides key lessons for other countries planning to apply for and implement a CCEOP grant.

### Guinea CCEOP Case Study

Over the last 20 years, immunization coverage in Guinea has remained low according to Demographic Health Survey data. The percentage of fully immunized children (i.e., children aged 12–23 months who received all basic vaccines) was only 24% in 2018.^10^ Fully immunized in this context means bacillus Calmette-Guerin; 3 doses of diphtheria, pertussis, tetanus, and *Haemophilus influenzae* type b; 3 doses of oral polio (not including the oral polio dose given at birth); and 1 dose of a measles-containing vaccine. There is also a marked variation in the proportion of fully immunized children among the country’s regions—from 8% in Labé to 36% in Kankan.^10^

Guinea has strived to improve vaccine service delivery by improving program coordination and increasing investment in the vaccine supply chain management system. Despite these efforts, challenges remain, including low human resource capacity in rural health centers, minimal cold storage capacity at the district and health center levels, poor accessibility of rural health centers, and poor data quality. Findings from the 2016 Effective Vaccine Management assessment illustrated the country’s shortcomings in meeting the standards in the 9 areas of effective vaccine management. The score for each criterion was well below the 80% minimum recommended by the World Health Organization (WHO).^11^

Despite Guinea’s efforts to improve vaccine service delivery, many challenges remain.

In 2016, Gavi approved a CCEOP grant of $10.9 million for Guinea to procure and install CCE in its health facilities. At the time of the CCEOP application, the primary level of the health system included 935 health posts and 410 health centers providing services that included vaccination. Health centers, typically with CCE, provided more frequent immunization services, while health posts, which lacked CCE, provided outreach immunization services (which require health workers to travel to collect vaccines from health centers equipped with CCE). With a focus on extending immunization services, the country prioritized installing equipment first at health facilities that had no CCE (mostly health posts) to increase the number of fixed places offering vaccinations. The second priority was to equip health centers that had nonfunctioning CCE. During the first deployment (December 2018–June 2019), 2 local SBPs installed a total of 848 pieces of equipment in the health posts. The second deployment was scheduled to take place between November 2021 and May 2022 but has been delayed.

#### Leadership and Country Ownership

Strong country leadership, provided largely by the PMT, was an important factor in CCEOP implementation and critical to the success of the first deployment in Guinea. The PMT, a subset and extension of the National Logistics Working Group (NLWG) and part of the EPI, consisted of a senior MOH adviser and representatives from EPI, SBP, technical partners (UNICEF and WHO), and other ministry entities such as finance and supply-chain management and maintenance.

Strong country leadership, provided largely by the PMT, was critical to the success of the first CCEOP deployment in Guinea.

Assessment findings at all stages of the evaluation show that the PMT played a major role in coordinating and planning the preparation and implementation of the CCEOP. In particular, the continuous exchange of ideas and problem solving backed by members’ technical expertise reinforced coordination between PMT members and all key stakeholders. Furthermore, regional-level respondents recognized the team for clearly communicating requirements during planning to all levels of the health system and providing monitoring and oversight when CCE was deployed. For example, the PMT visited a selected number of health posts to verify installation, training, functionality, and community awareness of newly available services due to the new CCE.

The EPI also exhibited strong leadership through its decisions. Early in the CCEOP application process (and before the establishment of the PMT), EPI prioritized investing in CCE at the health facilities that lacked it to increase the number of immunization services offered and improve coverage in the country. Later in the process and in consideration of the health system’s lack of capacity, the PMT recognized the value of using the SBPs to manage and conduct CCE distribution and installation (see human resource capacity section below). Per CCEOP guidance, the PMT and the UNICEF country office were responsible for approving SBP installation reports as a prerequisite for payment by UNICEF/Supply Division. However, despite their role in the monitoring and approval process, the evaluation highlighted the additional need for PMT and UNICEF country office review during development of the SBP contracts to better understand roles and support their oversight.

While leadership overall appeared to be strong during preparation, planning, and implementation of the first CCE deployment in Guinea, there was less active leadership in preparation for the second deployment. The NLWG was also less active than previously, and PMT members communicated on an as-needed basis but were otherwise inactive. They also were unable to conduct supervision activities or collect data on CCE inventory (due to data quality issues), facility needs, and other information, which delayed preparation for the second deployment. The coronavirus disease (COVID-19) pandemic also magnified delays as attention and resources were diverted to the national response.

#### Human Resource Capacity

Recognizing the limited human resource capacity within Guinea for CCE installation, many national-level stakeholders considered the SBP model to be highly efficient with faster and improved CCE installation. At the same time, they acknowledged the necessary tradeoff between the cost of contracting SBPs and efficiency. There was also concern about the implications of using SBPs on the sustainability of the national program.

Two SBPs in Guinea were selected to deliver, install, and provide after-sales service for the equipment and to train staff on operation and preventive maintenance. In addition to the repairs typically covered, 1 of the SBPs provided preventive maintenance for a percentage of the health posts over the life of the warranty. Within the CCEOP, this was an optional service the SBPs could provide for an additional fee as part of their overall contract. The SBP provision of preventive maintenance is a best practice because it focuses on prevention and highlights how SBPs can offer a menu of services tailored to countries’ needs.

As part of its contracts, Gavi also required the SBPs to provide training in CCE maintenance to ensure continuity after the warranties end. The SBPs conducted maintenance trainings for MOH and EPI staff and CCE technicians, and a high-level training/overview on preventive maintenance for facility-level staff with the expectation that health workers and technicians would conduct regular preventive and corrective maintenance. Subnational respondents noted that the effectiveness of these trainings varied. Issues noted by respondents included insufficient information in the trainings, differing training needs due to the varied education and skill levels of the trainees (i.e., the regional cold chain technicians vs. health facility staff), and turnover of staff trained. The MOH technicians also noted a lack of access to resources required to respond to maintenance requests for existing CCE, highlighting the need for training to sustain CCE and resources (i.e., human and financial capital) to conduct the maintenance (see sustainability section).

While these limitations jeopardize the country’s ability to ensure that CCE is adequately maintained post-CCE warranty, there were some promising practices. For example, in addition to training from the SBP, 2 of the 7 regional cold chain technicians participated in the CCE installation process and some also attended an additional training organized by EPI and facilitated by external technical experts.

#### Sustainability

CCEOP has injected a substantial investment into the cold chain system and the immunization program in Guinea. According to the endline evaluation, CCEOP improved country-level processes for equipment selection and installation, as well as components of the national management of the cold chain. Temperature monitoring data for the sampled facilities for the 60 days before endline data collection indicate optimally performing CCE within the required temperature range of 2°C–8°C for 95% of the time.

CCEOP improved country-level processes for equipment selection and installation, as well as components of national cold chain management.

**FIGURE 1:**
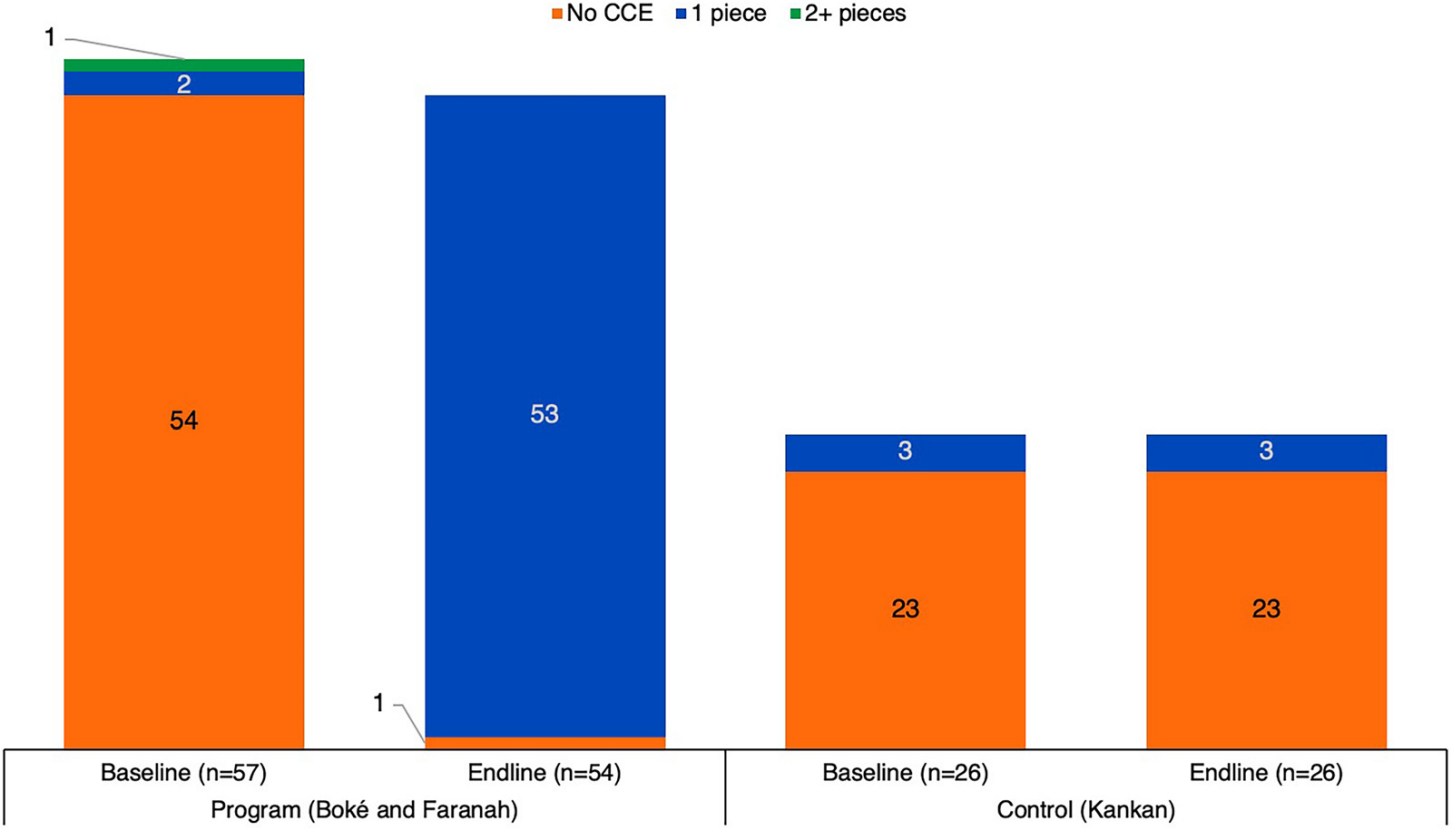
Number of Pieces of CCE in Health Posts in Guinea, by Region and Time Point Abbreviation: CCE, cold chain equipment.

**FIGURE 2:**
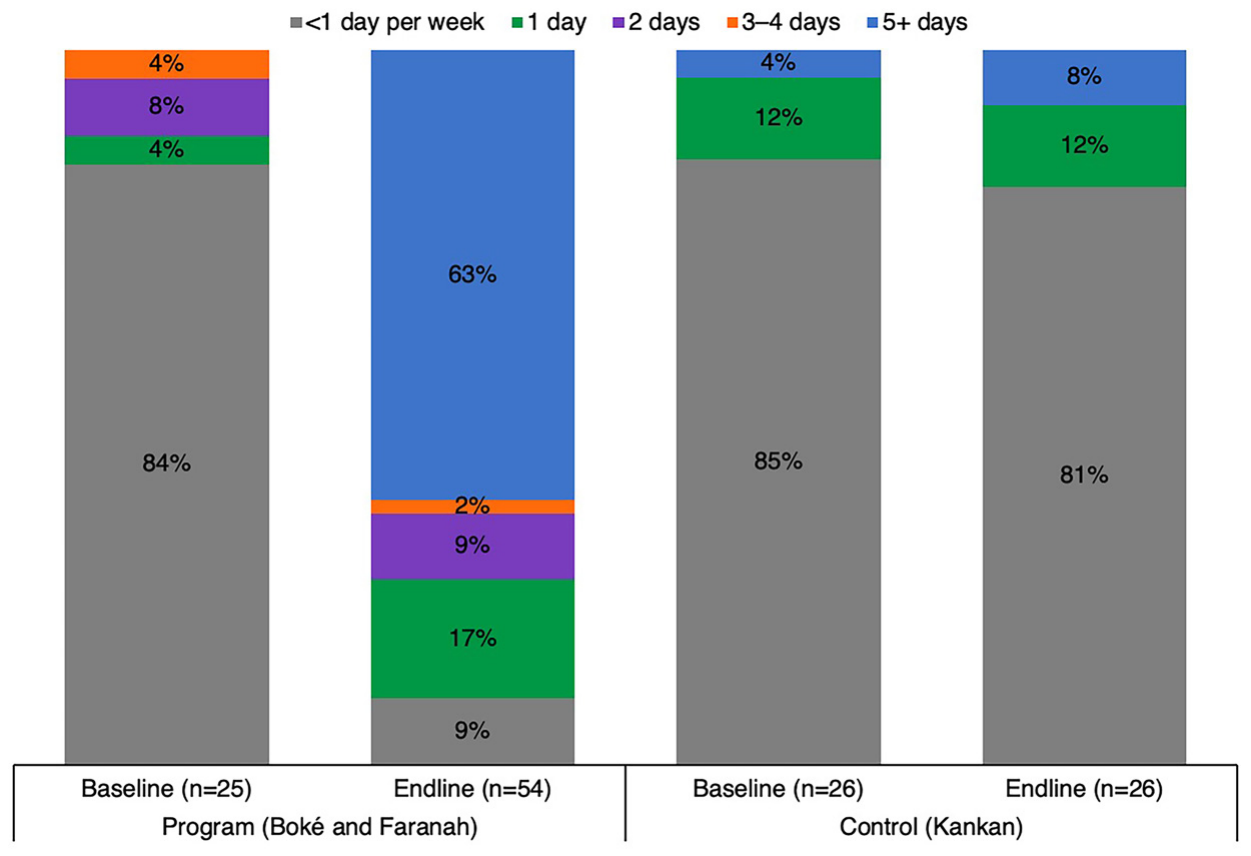
Frequency of Immunization Services Offered in Health Posts in Guinea (Days per Week), by Time Point^a^ ^a^Sample limited to heath posts offering in-facility routine immunization services.

Cold chain and immunization services have also been extended through CCEOP. Figure 1 shows the increase in availability of CCE among health posts in Boké and Faranah between baseline and endline (there was no change in Kankan). This has influenced the provision of immunization services in vaccinating facilities (Figure 2). At baseline, less than 50% of health posts in Boké and Faranah provided any immunization service. At endline, immunization service provision at these health posts more than doubled, while the proportion of services provided through outreach declined. Sixty-three percent of health posts offered immunization 5 or more days per week. This shift can be attributed to the new CCE installations, increased storage capacity to provide routine immunization services, and consequently a decreased need for outreach services. In the control region (Kankan) where CCE was not deployed, there was no change in immunization services offered between baseline and endline among the sampled health posts. Nearly all health posts in Kankan continued to provide services through outreach.

Sustaining these outcomes will be a challenge. In terms of maintaining CCE, which includes preventive maintenance for new equipment and corrective maintenance for new equipment when the warranties lapse, the government has taken several actions including adding a line item in the EPI budget to cover fuel, outreach transport for 1 year, and distribution of maintenance kits to technicians. However, stakeholders identified the need for more long-term solutions. For example, evaluation findings highlighted the need for organizational structural changes including a maintenance system that is adequately resourced with human capital and funding and has a reinvigorated leadership (i.e., PMT and EPI NLWG).

Stakeholders identified a need for more long-term solutions to sustaining CCE maintenance.

### Kenya CCEOP Case Study

According to the 2014 Kenya Demographic and Health Survey, approximately 75% of children aged 12–23 months are fully immunized. However, in remote and hard-to-reach areas, including counties in the Rift Valley and northeastern regions, as many as two-thirds of children are not fully immunized and therefore at risk of preventable life-threatening diseases.^12^

A 2016 national cold chain inventory shows that approximately 1 in 5 health facilities (18%) in Kenya did not have any CCE. Such facilities provide immunizations by collecting vaccines from those that do have CCE and using vaccine carriers and cold boxes for short periods.^13^ These facilities are most likely to offer reduced immunization services because they are unable to keep stock on hand.

Additionally, although 4 of 5 facilities had some type of CCE, the majority (81%) did not meet the WHO Performance, Quality, and Safety standards for the immunization supply chain.^13^ The results of Kenya's 2013 Effective Vaccine Management assessment demonstrated major limitations in almost all 9 key cold chain capacity domains of vaccine management, falling short of the minimum acceptable score of 80% on many of the domains.^14^

For these reasons, Gavi approved Kenya’s CCEOP application for US$8.2 million in March 2017 to provide CCE to health facilities and subcounty depots in 47 counties in multiple deployments (Table). The first deployment priorities focused on equipping facilities with an urgent need for CCE, while subsequent deployments were planned to focus on scale-up. The first deployment of equipment (1,004 refrigerators and freezers) to 690 health facilities and 291 subcounty depots was implemented according to schedule using local SBPs arranged by the CCE manufacturers. A planned second deployment has been delayed.

#### Leadership and Country Ownership

Leadership and country ownership in Kenya by experienced logistics professionals and knowledgeable and dedicated staff in the National Vaccines and Immunization Program (NVIP) were central to successful CCEOP implementation. The PMT leveraged experience from a variety of groups including NVIP and partners from the Clinton Health Access Initiative, WHO, and UNICEF. The PMT worked closely with the SBPs to monitor CCE deployment and installation and resolve any issues that could not be resolved at the county or subcounty levels. The PMT comprised a subset of individuals from the larger NLWG, a long-established group of individuals who represent a variety of government entities and partner organizations and advise on logistics-related immunization supply chain matters in Kenya. When individual PMT meetings became less frequent after the first equipment deployment, the NLWG took up and resolved many of the issues.

An engaged, subnational network of county EPI logisticians and cold chain technicians also contributed to a successful CCEOP implementation. While most of the CCEOP managing bodies were centrally staffed and managed, Kenya’s decentralized system also necessitated greater coordination with county and subcounty level staff. The cold chain inventory, required by Gavi to inform the application and comprehensive ODP, needed the input of county- and subcounty-level EPI logisticians and cold chain technicians. These individuals shared information about where CCE was located, its type and capacity, and the power sources at each facility, which informed the placement of new equipment through CCEOP. The county EPI logisticians also triaged requests from facilities and subcounties for maintenance or repairs on equipment and filtered these requests up to the MOH point of contact and then to the SBPs, who maintained and repaired eligible CCEOP equipment. An informal WhatsApp group facilitated rapid information exchange between county EPI logisticians, national staff, and the SBPs. This communication channel allowed subnational staff to reach SBPs more quickly and efficiently than if requests had to be sent by national EPI officials, which allowed SBPs to respond to and repair equipment under warranty in a more timely manner. As a result of these informal networks, in some cases, SBPs were able to troubleshoot equipment issues with a phone call or text to the county logistician or subcounty technician.

An engaged, subnational network of county EPI logisticians and cold chain technicians contributed to a successful CCEOP implementation.

The country also managed a much larger World Bank CCE deployment in parallel with CCEOP deployment that did not involve SBPs for installation or maintenance but that leveraged many of the same systems, like the cold chain inventory findings and the ODP requirement. The subnational network of EPI logisticians and cold chain technicians critically aided this effort, as well. However, despite the network’s engagement, findings from the assessment suggested that other subcounty-level staff, particularly clinical staff at facilities, were not engaged in the planning phases of CCEOP.

#### Human Resource Capacity

In Kenya, CCEOP implementation relied upon the extensive network of EPI logisticians and cold chain technicians throughout the country for equipment deployment and follow-up. Each county employs a lead cold chain technician who supervises the subcounty cold chain technicians at the subcounty levels. Two cold chain technicians from each of the 47 counties were brought to a central location for a training in May 2018, convened by UNICEF and NVIP. Over 2 weeks, the technicians received training on installation and preventive and corrective maintenance for the specific CCE models that were procured through CCEOP, even though these tasks would be performed by the SBPs during the equipment’s warranty period. Most participants, particularly those at the subnational level, reported that they were very satisfied with the training and noted that it also allowed them to install and maintain the same models of equipment procured through the World Bank, which did not come with the SBP bundles.

In many instances, the SBPs also provided basic preventive maintenance instruction to health facility staff when the equipment was installed. The installation team discussed how to clean the equipment, how frequently it should be defrosted, and how to reduce temperature excursions. The health facility staff appreciated these informal orientations but requested more formalized training with all staff, not just those who happened to be at the facility during installation.

#### Sustainability

**FIGURE 3:**
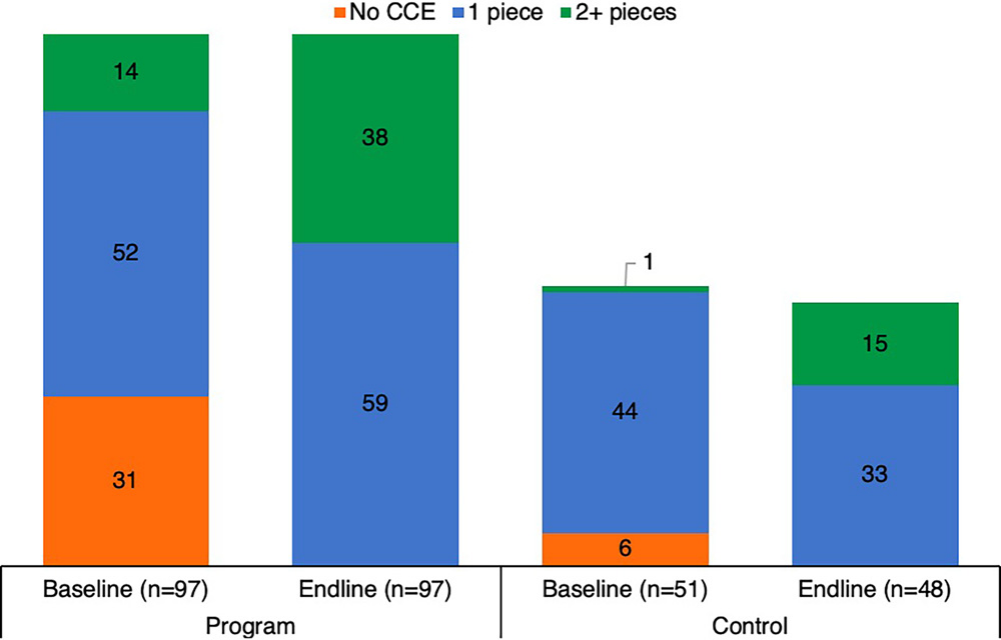
Number of Pieces of CCE in Each Facility in Kenya, by Study Arm and Time Point Abbreviation: CCE, cold chain equipment.

**FIGURE 4:**
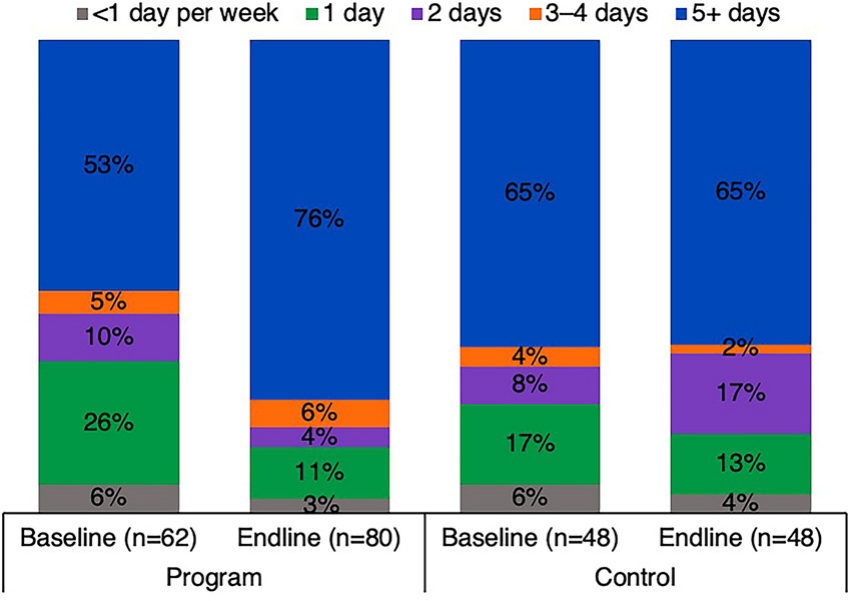
Frequency of Immunization Services Offered in Kenya (Days per Week), by Study Arm and Time Point

CCEOP has contributed to sustainability by greatly increasing the availability of CCE, which is newer and more reliable, as seen in Figure 3. At baseline, nearly one-third of the facilities in the program arm of the HFA sample had no CCE and had to collect vaccines from neighboring facilities on specific immunization days. At endline, all facilities had 1 or more pieces of CCE, extending the reach and expanding capacity to provide immunization services. This allowed immunization services to expand as well (Figure 4), with an increased proportion of facilities in the program arm of the HFA sample offering immunization services 3 or more days per week. Additionally, temperature reports from the CCE in the sampled facilities for the 60 days before data collection at endline show the equipment maintained temperatures in the required range for 98% of the time.

CCEOP has contributed to sustainability by greatly increasing the availability of newer, more reliable CCE.

Despite improvements in equipment availability and frequency of immunization services in program facilities, national-level respondents commonly noted the cost of the service bundles as the biggest barrier to CCEOP sustainability. Additionally, while satisfied with SBP installation of CCE, stakeholders at all levels expressed dissatisfaction with the lack of clarity related to maintenance services covered under the equipment warranties that were meant to be provided by the SBP postinstallation, particularly those of 1 manufacturer.

Due to the cost and the experience with some of the postinstallation SBP services, Kenya requested to eliminate (i.e., delink) the role of SBPs after the first deployment. While CCEOP would continue to be used as a procurement mechanism, the MOH would handle in-country equipment delivery, installation, and management. Gavi/UNICEF approved the request to delink, and many national-level respondents are confident that they have the capacity to manage the deployments moving forward. Additionally, managing the process themselves will develop the capacity of local cold chain technicians, which will contribute to the sustainability of the national cold chain system.

After the first deployment, Gavi/UNICEF approved Kenya’s request to handle future CCE delivery, installation, and management through the MOH.

However, in the absence of the SBPs, a robust maintenance system is required to sustain these improvements in immunization service frequency. Given Kenya’s decentralized nature, budgeting for maintenance systems takes place at the county level and varies greatly by county and competing priorities. For example, during the evaluation 1 county experienced maintenance budget shortfalls due to COVID-19 reallocation, which left equipment in need of repair for 6 months because there was no funding to transport it to the subcounty depot. Additionally, respondents from multiple counties noted that cold chain technicians who retired or quit were not replaced, resulting in gaps in human resources.

## DISCUSSION

The experiences of the Ministries of Health in Guinea and Kenya in deploying their CCEOP investments illustrate how countries prioritize and implement large funding and support mecha-nisms. As we examined the CCEOP journey thus far in Guinea and Kenya, several points surfaced that could apply to other countries planning to implement similar initiatives.

### Setting Priorities

When Guinea and Kenya were preparing their CCEOP applications, their needs differed based on their respective inventories and assessments. These needs, as well as Gavi guidance and priority standards, influenced the countries’ strategies and priorities for implementing and deploying the new equipment. For example, Guinea prioritized the CCE for facilities (mainly health posts) that did not have equipment. The goal was to extend immunization services to fixed sites that previously conducted outreach only and to increase immunization coverage. In comparison, Kenya’s priority was to accelerate CCE upgrading with new optimal equipment. In doing so, Kenya focused on expanding the functionality and capacity of cold chain devices in health facilities in most parts of the country.

### Partner Coordination

CCEOP is a multidimensional platform supporting numerous countries and yielding positive outcomes. To maximize efficiencies, this and other large initiatives require coordination and alignment with ministry systems and processes as well as those of other funder/partner initiatives. For example, in Kenya, efforts by multiple ongoing funders (World Bank, GIZ, Global Good) and partners (Clinton Health Access Initiative, WHO, UNICEF) made it essential for the CCEOP PMT to identify ways to work with them. In Guinea, fewer partners were operating, so the need for alignment did not appear to be notable. However, a 2018 Gavi joint assessment in Guinea noted that there is potential to better align coordination with other funders/partners to maximize the benefits of an optimal cold chain system.^15^ Establishing the PMT and building on the presence of NLWGs can help create a management structure to better coordinate all immunization supply chain partners and funders.

### SBP Innovations

The use of SBPs filled the essential capacity gap needed to install and maintain the CCE for the lifetime of the warranties. However, there are 4 important considerations if this model is to continue:

1. Contract management: The skills (including setting and managing key performance indicators) required to manage a third-party provider are often not considered when governments are deciding between performing specific functions in-house versus outsourcing them. As seen in Kenya, this includes making certain that those managing the SBPs have an understanding of the warranty agreements and what services are included and excluded as well as the ability and recourse to monitor and ensure compliance with warranty agreements.
2. Cost-effectiveness: The cost-effectiveness of each option is not often analyzed or considered. Frequently, a lack of data within the MOH systems complicates and hinders the ability to conduct a cost-effectiveness analysis. These estimates are required to understand the cost of performing these functions internally so that they can be compared with the cost of using a third-party provider.
3. Payment: Countries opting to use the SBP model often lack the mechanisms for timely guaranteed payments unless a funder or partner is paying the provider based on performance. As a result, service providers are hesitant to enter agreements.
4. Tailoring: To be most effective and contribute to sustainability, the SBP approach should be tailored to a country’s needs. For example, the SBP could be responsible for delivery to the facility level only, with the MOH responsible for installation. Another model could make the SBP responsible for installing more complex CCE, such as equipment requiring solar panels. The Kenyan MOH made the case to completely delink from the SBP based on its capacity to handle the process itself.

### A Systems Approach

Procurement of modern, upgraded, and efficient CCE is a critical precondition to strengthening the vaccine supply chain and improving immunization equity and coverage. Yet there are other equally important aspects, such as maintaining the CCE. Stakeholders in both countries expressed concern over the ongoing maintenance requirements, not just for the postwarranty period of CCEOP-procured equipment but also for equipment already in use. An inclusive, adequately resourced maintenance system will be increasingly important as the new CCE ages and requires more routine maintenance. In addition, better alignment with complementary activities could yield efficiency gains. Without addressing the operational and financial sustainability, the cold chain system that the immunization program relies on will depend on external funding. According to UNICEF, factors that disrupt the sustainable delivery of vaccine services include underinvestment in the national immunization program, among others.^3^ Therefore, investments in equipment should be considered part of a larger systems approach to improving immunization coverage.

Investments in equipment should be considered part of a larger systems approach to improving immunization coverage.

### System Efficiencies

The evaluation clearly showed that the offering of immunization services increased due to the availability of functional CCE. It is unclear, however, if increased services at a fixed place, such as a health center or post, have led or will lead to increased immunization coverage and equity. More studies should be conducted on the effectiveness of outreach services compared to those provided at a facility. At the same time, bringing additional CCE into the country has introduced efficiencies to the system as health workers no longer have to regularly collect vaccines from a higher-level facility or depots for outreach, because they now have a place to store them. Presumably, it has also brought the vaccines closer to the community in a more reliable way.

### Limitations

This case study has several limitations. While the evaluation included an HFA, the sample size was small, which limited calculation of statistical significance for differences across groups. In addition, due to the timing of the evaluation, the case study is limited to findings based on Deployment 1. An in-depth assessment of the full effect of CCEOP on immunization services and program sustainability would require a longer time frame.

## CONCLUSIONS

CCEOP provides a useful example of how large global health initiatives can strengthen MOH management systems while also investing in infrastructure, contributing to a more sustainable system. This article highlights how Guinea and Kenya demonstrated leadership and country ownership in prioritizing areas of weakness in the cold chain to address with CCEOP equipment, based on the country context. The processes established by Gavi, such as establishing the PMT, conducting a CCE inventory, and developing an ODP, strengthened the management structure and informed the development of best practices for these types of large health investments. These processes, combined with building human resource capacity, have contributed to a more sustainable cold chain system and immunization program. The experiences in Guinea and Kenya are applicable to countries embarking on similar investments. They are also relevant to agencies seeking to fund processes and standards that contribute to strengthening the overall health system.

## Translation

**En Français**

**UN CONTE DE 2 PAYS: MISE EN ŒUVRE DE LA PLATEFORME D'OPTIMISATION DES EQUIPEMENTS DE LA CHAINE DU FROID EN GUINEE ET AU KENYA**

**Messages clés**

- La mise en œuvre d'une intervention du système de santé impliquant la vaccination nécessite un haut niveau de leadership et d'appropriation par le pays, notamment avec des professionnels de la logistique expérimentés et un personnel compétent et spécialisé dans les programmes nationaux de vaccination et d'immunisation.
- Pour maximiser l'efficacité, la plateforme d'optimisation des équipements de la chaîne du froid de Gavi et d'autres initiatives de la sorte nécessitent une coordination et un alignement avec les systèmes et processus du ministère ainsi qu'avec ceux d'autres initiatives de bailleurs de fonds ou de partenaires.
- La sous-traitance d'une fonction spécifique comme l'installation d'équipements à un prestataire tiers peut être bénéfique pour les pays, mais les gouvernements doivent analyser le rapport coût-bénéfice de la sous-traitance à des prestataires ainsi que les compétences et les systèmes nécessaires pour assurer la gestion et la coordination avec ces derniers.
- Les équipements de la chaîne du froid renforcent la chaîne d'approvisionnement en vaccins et améliorent l'équité et la couverture vaccinales, mais leur pérennité en tant qu'investissement dans le système de santé dépend des ressources permanentes nécessaires à leur entretien. Si la viabilité opérationnelle et financière n'est pas assurée, les systèmes de chaîne du froid peu fiables et dépendants de financements extérieurs, ainsi que les programmes de vaccination qui en dépendent, ne pourront connaître aucune amélioration.

**RÉSUMÉ**

En 2016, la plateforme d'optimisation des équipements de la chaîne du froid (CCEOP) de Gavi a été approuvée et lancée, sachant que des équipements de la chaîne du froid (EEC) fonctionnels sont essentiels pour renforcer les chaînes d'approvisionnement en vaccins et, à terme, atteindre les objectifs d'équité et de couverture vaccinale de Gavi. Avec la plateforme CCEOP, Gavi s'est engagé à investir 250 millions de dollars US entre 2016 et 2021 pour mettre en service des équipements de la chaîne du froid dans plus de 63 000 établissements, afin d'améliorer et d'étendre l'implantation de ces équipements, tout en stimulant le marché pour offrir des équipements abordables, accessibles et à la pointe de la technologie. Nous présentons des études de cas de la Guinée et du Kenya, deux pays qui ont reçu le soutien de la plateforme CCEOP, qui mettent en évidence deux façons de prioriser les investissements dans les pays et de mettre en œuvre des activités par le biais d'un important mécanisme de financement et de soutien. Les études examinent les différentes manières dont chaque pays a mis en œuvre la plateforme CCEOP et analysent la manière dont les aspects de leadership et de capacité technique influencent les priorités et les résultats du pays. Elles permettent également de tirer des enseignements clés sur la durabilité d'un vaste effort dans le domaine de la chaîne d'approvisionnement en vaccins. Les exemples de la Guinée et du Kenya peuvent aider d'autres pays qui veulent lancer des interventions importantes similaires dans le domaine de la santé, notamment en ce qui concerne le renforcement de la chaîne d'approvisionnement et les programmes de vaccination. Ces exemples offrent en particulier des leçons importantes en matière de leadership, de processus et de systèmes, d'appropriation par le pays, de capacité technique et de durabilité.

**Peer Reviewed**

**First published online:** October 14, 2022.
